# The pediatric sepsis biomarker risk model

**DOI:** 10.1186/cc11652

**Published:** 2012-10-01

**Authors:** Hector R Wong, Shelia Salisbury, Qiang Xiao, Natalie Z Cvijanovich, Mark Hall, Geoffrey L Allen, Neal J Thomas, Robert J Freishtat, Nick Anas, Keith Meyer, Paul A Checchia, Richard Lin, Thomas P Shanley, Michael T Bigham, Anita Sen, Jeffrey Nowak, Michael Quasney, Jared W Henricksen, Arun Chopra, Sharon Banschbach, Eileen Beckman, Kelli Harmon, Patrick Lahni, Christopher J Lindsell

**Affiliations:** 1 Division of Critical Care Medicine, Cincinnati Children's Hospital Medical Center and Cincinnati Children's Research Foundation, Cincinnati, OH, USA; 2 Department of Pediatrics, University of Cincinnati College of Medicine, Cincinnati, OH, USA; 3 The EMD Millipore Corporation, St Charles, MO, USA; 4 Children's Hospital and Research Center Oakland, Oakland, CA, USA; 5 Nationwide Children's Hospital, Columbus, OH, USA; 6 Children's Mercy Hospital, Kansas City, MO, USA; 7 Penn State Hershey Children's Hospital, Hershey, PA, USA; 8 Children's National Medical Center, Washington, DC, USA; 9 Children's Hospital of Orange County, Orange, CA, USA; 10 Miami Children's Hospital, Miami, FL, USA; 11 Texas Children's Hospital, Houston, TX, USA; 12 The Children's Hospital of Philadelphia, Philadelphia, PA, USA; 13 CS Mott Children's Hospital at the University of Michigan, Ann Arbor, MI, USA; 14 Akron Children's Hospital, Akron, OH, USA; 15 Morgan Stanley Children's Hospital, Columbia University Medical Center, New York, NY, USA; 16 Children's Hospital and Clinics of Minnesota, Minneapolis, MN, USA; 17 Children's Hospital of Wisconsin, Milwaukee, WI, USA; 18 Primary Children's Medical Center, Salt Lake City, UT, USA; 19 St Christopher's Hospital for Children, Philadelphia, PA, USA; 20 Department of Emergency Medicine, University of Cincinnati College of Medicine, Cincinnati, OH, USA

## Abstract

**Introduction:**

The intrinsic heterogeneity of clinical septic shock is a major challenge. For clinical trials, individual patient management, and quality improvement efforts, it is unclear which patients are least likely to survive and thus benefit from alternative treatment approaches. A robust risk stratification tool would greatly aid decision-making. The objective of our study was to derive and test a multi-biomarker-based risk model to predict outcome in pediatric septic shock.

**Methods:**

Twelve candidate serum protein stratification biomarkers were identified from previous genome-wide expression profiling. To derive the risk stratification tool, biomarkers were measured in serum samples from 220 unselected children with septic shock, obtained during the first 24 hours of admission to the intensive care unit. Classification and Regression Tree (CART) analysis was used to generate a decision tree to predict 28-day all-cause mortality based on both biomarkers and clinical variables. The derived tree was subsequently tested in an independent cohort of 135 children with septic shock.

**Results:**

The derived decision tree included five biomarkers. In the derivation cohort, sensitivity for mortality was 91% (95% CI 70 - 98), specificity was 86% (80 - 90), positive predictive value was 43% (29 - 58), and negative predictive value was 99% (95 - 100). When applied to the test cohort, sensitivity was 89% (64 - 98) and specificity was 64% (55 - 73). In an updated model including all 355 subjects in the combined derivation and test cohorts, sensitivity for mortality was 93% (79 - 98), specificity was 74% (69 - 79), positive predictive value was 32% (24 - 41), and negative predictive value was 99% (96 - 100). False positive subjects in the updated model had greater illness severity compared to the true negative subjects, as measured by persistence of organ failure, length of stay, and intensive care unit free days.

**Conclusions:**

The pediatric sepsis biomarker risk model (PERSEVERE; PEdiatRic SEpsis biomarkEr Risk modEl) reliably identifies children at risk of death and greater illness severity from pediatric septic shock. PERSEVERE has the potential to substantially enhance clinical decision making, to adjust for risk in clinical trials, and to serve as a septic shock-specific quality metric.

## Introduction

In developed countries with ready access to powerful antibiotics and modern intensive care units, septic shock continues to be a major cause of morbidity and mortality in both adult and pediatric populations [1-4]. Experimental therapies continue to be evaluated. Yet, despite being based on sound biological principles and pre-clinical data, the majority of experimental therapies fail when tested in randomized, controlled trials [5]. While failure is likely multi-factorial, one consistent confounder is that septic shock is not a simple disease with uniform expression across a given patient cohort. Rather, septic shock is a complex syndrome displaying a tremendous degree of heterogeneity. It has been proposed that our inability to manage this heterogeneity is a major challenge for effective and rational clinical trials, and that a robust risk stratification tool could overcome this challenge [5,6].

We have been searching for biomarkers that might be associated with outcomes in pediatric septic shock using genome-wide expression profiling [7-17]. Through this discovery-oriented approach, we previously identified a panel of candidate stratification gene probes to predict outcome [18,19]. Twelve of these gene probes translate to readily measured serum protein biomarkers with known biological mechanisms suggesting a possible association with outcomes from septic shock. Our goal was to use these biomarkers to derive a risk stratification tool to identify those children with septic shock who are least likely to survive. Using classification and regression tree (CART) analysis, we derived and tested the Pediatric Sepsis Biomarker Risk Model (PERSEVERE).

## Materials and methods

### Patients, samples, and data collection

The study protocol was approved by the Institutional Review Boards of each of the 17 participating institutions. The data collection protocol was identical for both the derivation and test cohorts, and has been described in detail [12]. Briefly, children < 11 years of age admitted to a pediatric intensive care unit (PICU) and meeting pediatric-specific criteria for septic shock were eligible [20]. Full-term neonates (that is, < 28 days of age) re-admitted to the hospital for septic shock were included. Clinical care was not directed by the study, and except for when informed consent could not be obtained, no child was excluded. After informed consent was obtained from parents or legal guardians, and within 24 hours of admission to the PICU, serum samples were obtained. Annotated clinical and laboratory data were collected daily while the participant was in the PICU. Illness severity was prospectively calculated using the pediatric risk of mortality (PRISM) score [21]. The number of organ failures during the initial 7 days of PICU admission was recorded using pediatric-specific criteria [20]. PICU-free days were calculated by subtracting the actual PICU length of stay (LOS) from a theoretical maximum PICU LOS of 28 days. Patients with a PICU LOS greater than 28 days were classified as having zero PICU-free days. The primary outcome variable was all-cause 28-day mortality.

### Candidate biomarkers

The 12 candidate biomarkers (gene symbols) included: C-C chemokine ligand 3 (CCL3), C-C chemokine ligand 4 (CCL4), neutrophil elastase 2 (ELA2), granzyme B (GZMB), heat shock protein 70 kDa 1B (HSPA1B), interleukin 1α (IL1A), interleukin 8 (IL8), lipocalin 2 (LCN2), lactotransferrin (LTF), matrix metalloproteinase 8 (MMP8), resistin (RETN), and thrombospondin 1 (THBS1). These were selected from 117 gene probes demonstrating outcome predictive strength in microarray-based studies involving children with septic shock [18,19]. The serum concentrations of the candidate biomarkers were measured using a multiplex magnetic bead platform (MILLIPLEX™ MAP) designed for this project by the EMD Millipore Corporation (Billerica, MA, USA). Biomarker concentrations were measured in a Luminex^® ^100/200 System (Luminex Corporation, Austin, TX, USA), according to the manufacturers' specifications. Assay performance data are provided in Additional File 1.

### Statistical analysis

Initially, data were described using medians, interquartile ranges (IQR), frequencies, and percents. Comparisons between survivors and non-survivors were performed using the Mann-Whitney *U*-test, Chi-square test, or Fisher's Exact test as appropriate. Analysis of descriptive statistics and comparisons were performed using SigmaStat Software (Systat Software, Inc., San Jose, CA, USA).

To derive the decision tree, we employed a classification and regression tree (CART) approach for the determination of biomarker cutoffs [22]. All 12 candidate biomarkers, as well as age and gender were considered in the CART analysis. The classification tree was built using Salford Predictive Modeler v6.6 (Salford Systems, San Diego, CA, USA). The model parameters, pruning criteria, and the command file for reproducing the classification tree are provided in Additional File 2. Performance of the decision tree is reported using diagnostic test statistics with 95% confidence intervals computed using the score method as implemented by VassarStats Website for Statistical Computation [23].

## Results

### Derivation of PERSEVERE

The demographics and clinical characteristics of the derivation cohort (n = 220) are provided in Table 1. The 23 (10.5%) non-survivors had a higher median PRISM score compared to the 197 survivors. Age, gender, race, infection characteristics, and occurrence of comorbidities did not differ significantly between survivors and non-survivors. The mean ± SD and median (IQR) times to death in the derivation cohort non-survivors were 6.1 ± 7.5 and 2 (1 to 8) days, respectively. A complete list of comorbidities for the survivors in the derivation cohort is provided in Additional File 3. A list of causative organisms for the derivation cohort is provided in Additional File 4.

**Table 1:** Demographics and clinical characteristics of the derivation cohort

	All	Survivors	Non-survivors
**Number of subjects**	220	197	23
**Median age in years (25%, 75%)^1^**	2.2 (0.8, 5.9)	2.3 (1.0, 5.9)	1.4 (0.2, 4.2)
**Median PRISM score (25%, 75%)**	15 (8, 22)	13 (7, 20)	28 (20, 37)^2^
**Number of males (%)**	137 (62)	120 (61)	17 (74)
**Number of females (%)**	83 (38)	77 (39)	6 (26)
**Number for race (%)** **Caucasian** **African American** **Other^3^** **Unreported**	153 (70) 39 (18) 12 (5) 16 (7)	138 (70) 35 (17) 11 (6) 13 (7)	15 (65) 4 (17) 1 (4) 3 (13)
**Number with gram (+) bacteria (%)**	70 (32)	61 (31)	9 (39)
**Number with gram (-) bacteria (%)**	55 (25)	51 (26)	4 (17)
**Number with viral infection (%)**	16 (7)	13 (7)	3 (13)
**Number with fungal infection (%)**	7 (3)	6 (3)	1 (4)
**Number with no organism isolated (%)**	72 (33)	66 (34)	6 (26)
**Number with any co-morbidity (%)**	91 (41)	82 (42)	9 (39)^4^
**Number with meningitis (%)**	12 (5)	10 (5)	2 (9)
**Number with cancer (%)**	17 (5)	17 (9)	0 (0)
**Number with immune suppression (%)^5^**	16 (7)	13 (7)	3 (13)

^1^Two subjects in the derivation cohort were older than stated in the protocol (13 and 14 years of age) but were included in the analysis. ^2^*P *< 0.001 vs. survivors. ^3^Includes Asian, multi-racial, native Hawaiian/Pacific Islander, and American Indian. ^4^Co-morbidities in non-survivors included anti-phospholipid antibody syndrome; aplastic anemia; chronic lung disease (2 subjects); DiGeorge Syndrome; developmental delay (2 subjects); hypoplastic left heart syndrome; and short gut syndrome. ^5^Refers to patients with immune suppression not related to cancer (for example, those receiving immune suppressive medication for solid organ transplantation, or those with a primary immune deficiency).

**Figure 1:**
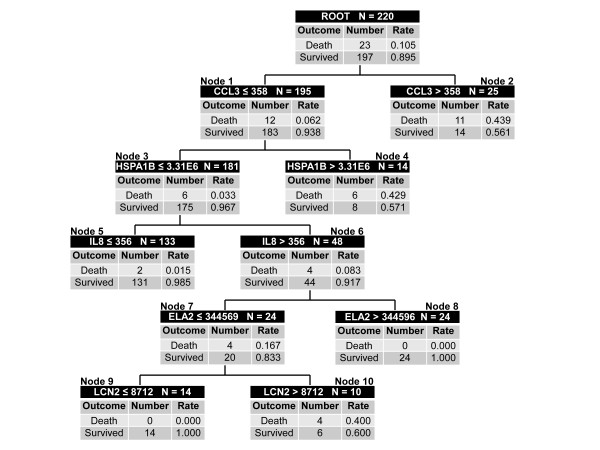
**Classification tree from the derivation cohort (n = 220)**. The classification tree consists of five biomarker-based decision rules and ten daughter nodes. The classification tree includes five of the twelve candidate stratification biomarkers: C-C chemokine ligand 3 (CCL3), heat shock protein 70 kDa 1B (HSPA1B), interleukin-8 (IL8), elastase 2 (ELA2), and lipocalin 2 (LCN2). Each node provides the total number of subjects in the node, the biomarker serum concentration-based decision rule, and the number of survivors and non-survivors with the respective rates. For consistency, the serum concentrations of all stratification biomarkers are provided in pg/ml. Terminal nodes 5, 8, and 9 are considered low-risk nodes, whereas terminal nodes 2, 4, 10 are considered high-risk terminal nodes. To calculate the diagnostic test characteristics, all subjects in the low-risk terminal nodes (n = 171) were classified as predicted survivors, whereas all subjects in the high-risk terminal nodes (n = 49) were classified as predicted non-survivors. The area under the curve (AUC) for the derivation cohort tree was 0.885.

The derived decision tree is shown in Figure 1. Maximum accuracy was achieved with five of the twelve candidate stratification biomarkers: CCL3, HSPA1B, IL8, ELA2, and LCN2. No demographic or clinical variables improved predictive accuracy. There were three low-risk terminal nodes (≤ 1.5% risk of death; nodes 5, 8, and 9) and three high-risk terminal nodes (≥ 40% risk of death; nodes 2, 4, and 10). Of the 171 participants classified as low-risk, 169 survived and 2 (1.2%) had died by 28 days. Of the 49 participants classified as high risk, 21 (42.9%) had died by 28 days. The diagnostic test characteristics are shown in Table 2.

**Table 2:** Performance of the classification trees

	Derivation cohort	Test cohort	Updated model
**Number of subjects**	220	135	355
**Number of true positives**	21	16	38
**Number of true negatives**	169	75	233
**Number of false positives**	28	42	81
**Number of false negatives**	2	2	3

**Sensitivity**	91% (70, 98)	89% (64, 98)	93% (79, 98)

**Specificity **	86% (80, 90)	64% (55, 73)	74% (69, 79)
**Positive predictive value**	43% (29, 58)	28% (17, 41)	32% (24, 41)
**Negative predictive value**	99% (95, 100)	97% (90, 100)	99% (96, 100)
**+Likelihood ratio**	6.4 (4.5, 9.3)	2.5 (1.8, 3.3)	3.6 (2.9, 4.4)
**-Likelihood ratio**	0.1 (0.0, 0.4)	0.2 (0.0, 0.6)	0.1 (0.0, 0.3)
**Area under the curve**	0.885	0.759	0.883

### Testing PERSEVERE

The independent test cohort consisted of 135 participants with septic shock, of whom 18 (13.3%) did not survive to 28 days. Table 3 provides the demographic and clinical characteristics of the test cohort. Compared to the derivation cohort, the test cohort had a higher proportion of Caucasians and a greater proportion with no causative organism isolated. The test cohort also had a lower proportion with no reported race and a lower proportion with gram-positive bacteria, compared to the derivation cohort. The test and derivation cohorts were otherwise not statistically different. Within the test cohort, there were no significant differences between survivors and non-survivors, except for the median PRISM scores. The mean and median times to death in the test cohort non-survivors were 9.9 ± SD 11.2 and 4 (IQR 2 to 16) days, respectively. A complete list of comorbidities for the survivors in the test cohort is provided in Additional File 3. A list of causative organisms for the test cohort is provided in Additional File 4.

**Table 3:** Demographics and clinical characteristics of the test cohort

	All	Survivors	Non-survivors
**Number of subjects**	135	117	18

**Median age in years (25%, 75%)**	2.8 (1.0, 6.7)	2.7 (1.0, 6.7)	3.8 (0.9, 7.7)

**Median PRISM score (25%, 75%)**	13 (7, 19)	12 (7, 18)	23 (14, 32)^1^

**Number of males (%)**	70 (52)	63 (54)	7 (39)

**Number of females (%)**	65 (48)	54 (46)	11 (61)
**Number for race (%)**			
**Caucasian**	113 (84)^2^	99 (85)	14 (78)
**African American**	15 (11)	13 (11)	2 (11)
**Other^3^**	6 (4)	4 (3)	2 (11)
**Unreported**	1 (1)^2^	1 (1)	0 (0)

**Number with gram (+) bacteria (%)**	27 (20)^2^	24 (21)	3 (17)

**Number with gram (-) bacteria (%)**	27 (20)	22 (19)	5 (28)

**Number with viral infection (%)**	10 (7)	9 (8)	1 (6)

**Number with fungal infection (%)**	2 (1)	2 (2)	0 (0)

**Number with no organism isolated (%)**	72 (53)^2^	63 (54)	9 (50)

**Number with any co-morbidity (%)**	52 (39)	45 (38)	7 (39)^4^

**Number with meningitis (%)**	5 (4)	3 (3)	2 (11)

**Number with cancer (%)**	17 (13)	14 (12)	3 (17)

**Number with immune suppression (%)^5^**	13 (10)	13 (11)	0 (0)

^1^*P *= 0.001 vs. survivors. ^2^*P *<0.05 for test cohort vs. derivation cohort. ^3^Includes Asian, multi-racial, native Hawaiian/Pacific Islander, and American Indian. ^4^Co-morbidities in non-survivors included acute myeloid leukemia; atrial and ventricular septal defects; fulminant hepatic failure; hypoplastic left heart syndrome; short gut syndrome; neuroblastoma; and optic nerve glioma. ^5^Refers to patients with immune suppression not related to cancer (for example, those receiving immune suppressive medication for solid organ transplantation, or those with a primary immune deficiency).

The classification of the test cohort participants according to the decision tree is shown in Additional File 5. Seventy-seven patients were classified as low risk (nodes 5 and 8), while 58 were classified as high risk (nodes 2, 4, and 10). Among the low-risk participants, the mortality rate was 2.6%, while among the high-risk participants the mortality rate was 27.6%. The diagnostic test characteristics of the decision tree in the test cohort are shown in Table 2.

### Secondary considerations

**Figure 2:**
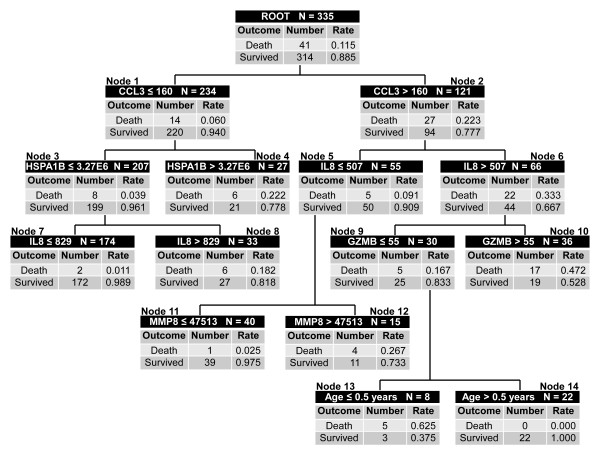
**Classification tree from the updated model based on the combined derivation and test cohorts (n = 355)**. The classification tree consists of six biomarker-based decision rules, one age-based decision rule, and fourteen daughter nodes. The classification tree includes five of the twelve candidate stratification biomarkers: C-C chemokine ligand 3 (CCL3), heat shock protein 70 kDa 1B (HSPA1B), interleukin-8 (IL8), granzyme B (GZMB), and matrix metalloproteinase-8 (MMP8). Each node provides the total number of subjects in the node, the biomarker serum concentration- or age-based decision rule, and the number of survivors and non-survivors with the respective rates. For consistency, the serum concentrations of all stratification biomarkers are provided in pg/ml. Terminal nodes 7, 11, and 14 are considered low-risk nodes, whereas terminal nodes 4, 8, 10, 12, and 13 are considered high-risk terminal nodes. To calculate the diagnostic test characteristics, all subjects in the low risk terminal nodes (n = 236) were classified as predicted survivors, whereas all subjects in the high risk terminal nodes (n = 119) were classified as predicted non-survivors. The area under the curve (AUC) for the re-calibrated decision tree was 0.883.

The classification tree was updated using all 355 participants in the combined derivation and test cohorts. The model parameters, pruning criteria, and the command file for reproducing the updated classification tree are provided in Additional File 6. All 12 candidate biomarkers, as well as age and gender were considered in the updating process. The updated decision tree is shown in Figure 2. Maximum accuracy was achieved with three of the same stratification biomarkers (CCL3, HSPA1B, and IL8), while the importance of ELA2 and LCN2 were superseded by GZMB and MMP8. Age also added to the predictive capacity of the updated tree (nodes 13 and 14). There were three low-risk terminal nodes (0.0 to 2.5% risk of death; nodes 7, 11, and 14) and five high-risk terminal nodes (18.2 to 62.5% risk of death; nodes 4, 8, 10, 12, and 13). Of the 236 participants classified as low risk, 233 survived (98.7%) and 3 had died (1.3%) by 28 days. Of the 119 participants classified as high risk, 38 had died (31.9%) by 28 days. The diagnostic test characteristics of the updated decision tree are shown in Table 2.

If PERSEVERE is clinically relevant and biologically plausible, then one could postulate that the 81 false-positive participants in the updated decision tree (that is, Those predicted to be non-survivors, but were actually survivors) would demonstrate an increased degree of organ dysfunction and PICU LOS, and less PICU-free days, compared to the 233 true-negative participants (that is, those predicted to be survivors and were actually survivors). Thirty percent of the false-positive participants had persistence of two or more organ failures at 7 days after study entry, compared to only 9% of the true-negative participants (*P *< 0.001). The median (IQR) PICU LOS for the false positive participants was 11 (6 to 17) days, compared to 7 (4 to 12) days for the true-negative participants (*P *= 0.003). Additionally, 64% of the false-positive participants had a PICU LOS > 1 week, compared to 46% of the true-negative participants (*P *= 0.01). The median PICU-free days for the false-positive participants was 18 (12 to 23) days, compared to 21 (16 to 25) days for the true-negative participants (*P *= 0.006). Additionally, 58% of the false-positive participants had < 21 PICU-free days, compared to 44% of the true-negative participants (*P *= 0.025).

## Discussion

We have derived and tested a biomarker-based risk stratification tool, PERSEVERE, which appears to reliably predict mortality in children with septic shock. PERSEVERE was derived using CART analysis, a potentially powerful approach for discovering complex predictor variable interactions that may not be evident using more traditional approaches [22,24]. The major drawback of CART analysis is the potential to over-fit a given dataset. Consequently, it is imperative that CART-derived models be tested using an independent dataset. When we applied PERSEVERE to an independent cohort of children with septic shock, those predicted as non-survivors had more than 25% mortality by 28 days. Additionally, the high-risk survivors in the updated model were found to have a greater degree of illness severity as measured by persistence of organ failure, PICU LOS, and PICU-free days.

A major strength of PERSEVERE is the initial approach to deriving the candidate biomarkers [18,19]. Using our extensive genome-wide expression databank, we objectively selected 12 of the 117 gene probes possibly associated with outcome in a cohort of children with septic shock. The selection criteria defined a priori were that: 1) the gene product (that is, protein) must have biological and mechanistic plausibility regarding the host response to infection, immunity, and/or inflammation, and 2) the gene product must be capable of being readily measured in the serum compartment. Accordingly, the selection of the candidate biomarkers was a relatively unbiased process, although we recognize some might consider assertion of biological and mechanistic plausibility and the technical limitation of being able to measure the protein in the serum compartment as causes of bias.

Another strength of PERSEVERE is its potential for generalizability. The PERSEVERE derivation and test cohorts represent 17 different institutions throughout the USA. Participant eligibility was unrestricted and enrollment was based exclusively on pediatric-specific criteria for septic shock. The only exclusion criterion was the inability to obtain informed consent. Consequently, the study cohorts represent the entire spectrum of pediatric septic shock, including patients with a broad range of significant co-morbidities typically encountered in clinical practice. In addition, the mortality rate and illness severity in this study are consistent with published studies [4,25,26]. Because clinical care was not under protocol, PERSEVERE appears to be independent of variability in local clinical practice patterns and nuances. We contend that these features will allow for feasible application of PERSEVERE in clinical practice.

We are not aware of a validated stratification tool for pediatric septic shock that performs in an equivalent manner to that of PERSEVERE. We previously proposed and tested a cutoff value for serum IL8 having a 95% negative predictive value (NPV) for mortality in pediatric septic shock [27]. However, the sensitivity, specificity, and positive predictive value (PPV) of IL8 in isolation were substantially lower than that of PERSEVERE. Three biomarkers (CCL3, HSPA1B, and IL8) appear to be the primary predictors in PERSEVERE. These three biomarkers consistently contribute to the upper level decision rules of both the initially derived tree and the subsequent updated tree. The lower-level decision rules appear to be less clear. ELA2 and LCN2 contributed to predictive capacity in the initially derived tree, but not in the subsequent updated tree, which instead included GZMB, MMP8, and age. Notably, members of our group are currently pursuing GZMB [28,29] and MMP8 [14] as novel therapeutic targets in septic shock, and younger age was previously linked to higher mortality in pediatric septic shock [4]. We expect that including additional patients in future modeling procedures will further define the components of the lower-level decision rules.

Illness severity scores (such as PRISM) are robust for predicting the outcome of general ICU populations, but are not intended for stratification and are not septic shock-specific [30]. Nonetheless, we expect there will be interest in comparing PERSEVERE performance with that of PRISM. As shown in Additional File 7, the updated model has a higher area under the curve than PRISM. In addition, at a comparable sensitivity of 93%, the PPV and specificity of PERSEVERE are 2-fold higher than that of PRISM.

An overall 32% PPV for mortality in the updated model may be viewed as being relatively low. However, PPV is highly influenced by prevalence and consequently needs to be interpreted in the context of prevalence [19]. In our study cohort, overall mortality was 11%. Therefore, the model identifies a cohort (namely, high-risk patients) with a mortality rate that is almost 3-fold higher than the overall cohort mortality. In addition, the model identifies a cohort (namely, low-risk patients) with an overall morality of 1%. Thus, at its most basic level, PERSEVERE divides the overall cohort into two populations having a 30-fold difference in mortality.

We envisage several applications of PERSEVERE. First, it could be used to select participants for interventional clinical trials. Excluding participants with very low mortality risk, while simultaneously selecting those at greatest mortality risk, increases the magnitude of possible survival benefit of a new therapy, while not placing those most likely to survive at risk of any adverse effects of a new therapeutic approach. Based on the test characteristics of the updated model, PERSEVERE has the potential to exclude patients, having up to a 99% probability of survival with standard care, and include patients with up to a 32% probability of death. The latter is clinically relevant given that the best available epidemiological data indicate an overall mortality of about 10% for pediatric septic shock in the USA [1,4]. The largest pediatric septic shock interventional trial to date employed a surrogate primary outcome variable because power calculations based on an assumed mortality rate of 12% would have required more than 3,000 subjects to achieve sufficient power to detect an absolute decrease in mortality of 2% [25]. Beginning with a cohort at higher predicted risk of mortality would have allowed greater flexibility in study design, with the target of a larger absolute risk reduction, and hence a smaller sample size. By stratifying patients via PERSEVERE, one has the potential to optimize the risk-to-benefit ratio of a test agent having more than minimal risk, and consequently conduct more rational clinical trials. Importantly, PERSEVERE was developed using serum collected during the first 24 hours of admission to the PICU, which is the optimal period for initiating new therapeutic approaches, and thus for risk-stratifying patients. If PERSEVERE is not used to determine eligibility, it could be taken into account by conducting a stratified outcomes analysis.

Outside of the clinical trial context, PERSEVERE could help inform clinical decisions regarding the application of high risk, invasive therapeutic and support modalities in septic shock, such as extracorporeal life support, plasmapheresis, and pulmonary artery catheterization. Finally, PERSEVERE has the potential to serve as a benchmark for septic shock-specific quality improvement and quality assurance efforts. For example, based on the updated model, higher than 1% mortality in the lowest-risk patients might be an indicator of poor performance, while lower than 32% mortality in the highest-risk group might be indicative of good performance. Moreover, differences in illness severity in those who survived but who were predicted to die, and in those who survived and were predicted to survive, could provide some clues to tailoring treatments to improve outcomes for all pediatric septic shock patients.

We envisage that PERSEVERE will be dynamic and require periodic updating. As we include more patients into the modeling process, some of the biomarker cutoff values that drive the decision tree may change. It is also possible that new biomarkers are identified that might contribute to the decision tree, or that previously tested biomarkers might be useful for refining the risk stratification. This evolution would enhance predictive performance and further increase generalizability of the decision tree. These assertions are evident in the updating process involving the combined derivation and test cohorts. Finally, we also envisage that PERSEVERE could provide the foundation for deriving an analogous stratification model for adult septic shock.

A potential weakness of PERSEVERE is that it is focused on relatively short-term outcomes and does not evaluate longer-term outcomes. Twenty-eight day all-cause mortality has been a standard primary outcome measure for multiple septic shock interventional clinical trials, but its usefulness has been questioned and there is increasing recognition that septic shock has significant negative consequences for quality of life beyond the dichotomy of the patient being alive or dead at 28 days [1,5]. The PERSEVERE protocol was not specifically designed to assess longer-term outcomes. However, the increased illness severity found in the false-positive patients, compared to the true-negative patients, indirectly suggests that PERSEVERE may have the capability of stratifying longer-term outcomes, but this assertion requires formal testing. We maintain that 28 day mortality remains a clinically relevant outcome variable in clinical septic shock; at the very least, one must be alive beyond 28 days in order to assess longer-term outcomes.

Another potential weakness of our study is that it is difficult to unambiguously assign septic shock as the primary cause of death in all non-survivors. However, all-cause mortality is a common outcome variable in septic shock clinical trials, and the distribution of co-morbidities across the survivors and non-survivors indirectly suggests that many of the deaths could be, at least partially, attributed to septic shock per se.

## Conclusions

We have derived and successfully tested a biomarker-based risk model to stratify pediatric septic shock outcome. The basis of the model is a high throughput, relatively unbiased, microarray-based approach, and the derivation and test cohorts well represent the intrinsic heterogeneity and broad spectrum of pediatric septic shock encountered in clinical practice. We propose that PERSEVERE has the potential to substantially enhance the conduct of clinical trials, inform clinical decision making, and serve to inform septic shock-specific quality improvement measures.

## Key messages

• We have derived a multi-biomarker-based risk model to predict outcome in pediatric septic shock.

• The derived model has been successfully tested in a separate cohort.

• An updated model has a PPV for mortality of 32% and a NPV of 99%, thus generating two cohorts having a 30-fold difference in mortality.

• False-positive patients have a greater level of illness severity than true-negative patients as measured by organ failure, length of stay, and ICU-free days.

• The risk model has potential applications for clinical trial stratification, individual patient decision making, and quality assurance efforts.

## Abbreviations

AUC: area under the curve; CART: classification and regression tree; CCL: C-C chemokine ligand; ELA: neutrophil elastase; GZMB: granzyme B; HSPA1B: heat shock protein 70 kDa 1B; LCN: IQR: interquartile range; lipocalin; LOS: length of stay; LTF: lactotransferrin; MMP: matrix metalloproteinase; NPV: negative predictive value; PERSEVERE: Pediatric Sepsis Biomarker Risk Model; PICU: pediatric intensive care unit; PPV: positive predictive value; PRISM: pediatric risk of mortality; RETN: resistin; THBS: thrombospondin.

## Competing interests

HRW and the Cincinnati Children's Hospital Research Foundation have submitted a provisional patent application for PERSEVERE. SS is named as a co-inventor in the above patent application. QX is an employee of the EMD Millipore Corporation. CJL is named as a co-inventor in the above patent application. The remaining authors have no competing interests to report.

## Authors' contributions

HRW conceived and developed the study, obtained funding for the study, directly took part in the analyses, and wrote the manuscript. SS conducted the statistical analyses, assisted in obtaining funding, and edited the manuscript. QX developed the multiplex platform to measure the candidate serum protein stratification biomarkers. NZC, MH, GLA, NJT, RJF, NA, KM, PAC, RL, TPS, MTB, AS, JN, MQ, JH, and AC enrolled patients, and provided biological samples and clinical data for the database. SB and EB *c*oordinated patient enrollment among the various study sites and maintained the clinical database. KM coordinated biological sample procurement and submission among the various study sites, and maintained the biological specimen repository. PL conducted all biomarker measurements. CJL collaborated in the initial design of the study and in obtaining funding, oversaw the statistical analyses, and edited the manuscript. All authors read and approved the final manuscript.

## Supplementary Material

Additional File 1 **Performance data for the biomarker assays**. This file provides technical data regarding the development of the biomarker assays. Click here for file

Additional File 2 **Derivation of the classification tree using Salford Predictive Modeler v6.6**. This file provides the model parameters, pruning criteria, and the command file for generating the decision tree. Click here for file

Additional File 3 **List of comorbidities in survivors for the derivation and test cohorts**. This file contains Table S1, which provides a list of co-morbidities for patients (survivors) in the derivation and test cohorts. Click here for file

Additional File 4 **Causative organisms for derivation and test cohorts**. This file contains Table S2, which provides a list of causative organisms for patients in the derivation and test cohorts. Click here for file

Additional File 5 **Figure S1 that demonstrates the classification of the test cohort patients according to the derived decision tree**. Classification tree for the test cohort (n = 135). The biomarker-based decision rules from the derivation cohort tree were applied to the test cohort with no modifications. The same conventions that were applied to the derivation cohort for calculating diagnostic test characteristics were applied to the test cohort, except that no patients in the test cohort occupied low risk terminal node 9. The area under the curve for the test cohort tree was 0.759. Click here for file

Additional File 6 **Updating the classification tree using Salford Predictive Modeler v6.6**. This file provided the model parameters, pruning criteria, and the command file for generating the updated decision tree. Click here for file

Additional File 7 **Comparison of PERSEVERE and PRISM for predicting mortality in the combined derivation and test cohorts**. This file contains Table S3, which compares the test characteristics of PERSEVERE and PRISM. Click here for file
