# Supplementary material for: The pediatric sepsis biomarker risk model
Source: Crit Care. 2012 Oct 1;16(5):R174. doi: 10.1186/cc11652 (PMC3682273; doi:10.1186/cc11652)
Supplement: Additional File 1 — Performance data for the biomarker assays. This file provides technical data regarding the development of the biomarker assays. [file cc11652-S1.DOC]

**ADDITIONAL FILE 1:** Performance data for the biomarker assays.

PANEL 1: GZMB, HSPA1B, IL1A, IL8, CCL3, CCL4, and MMP8

SENSITIVITY (N = 8 ASSAYS)

| **Analyte** | **Minimum Detectable Concentration (pg/ml)** | **Minimum Detectable Concentration + 2 S.D.** |
| --- | --- | --- |
| GZMB | 0.8 | 1.3 |
| HSPA1B | 2,930 | 4438 |
| IL1A | 2.6 | 4.8 |
| IL8 | 1.1 | 2.5 |
| CCL3 | 2.0 | 4.9 |
| CCL4 | 2.6 | 5.4 |
| MMP8 | 65 | 162 |

INTRA- AND INTER-ASSY % COEFFICIENTS OF VARIATION (%CV)

| **Analyte** | **Intra-assay %CV** | **Inter-assay %CV** |
| --- | --- | --- |
| GZMB | 7 | 8 |
| HSPA1B | 9 | 10 |
| IL1A | 8 | 7 |
| IL8 | 5 | 14 |
| CCL3 | 4 | 11 |
| CCL4 | 7 | 9 |
| MMP8 | 7 | 10 |

SPIKE RECOVERY IN SERUM MATRIX SOLUTION

| **Analyte** | **% Recovery in Serum Matrix** |
| --- | --- |
| GZMB | 95 |
| HSPA1B | 92 |
| IL1A | 97 |
| IL8 | 98 |
| CCL3 | 92 |
| CCL4 | 87 |
| MMP8 | 92 |

PANEL 2: ELA2, LTF, LCN2, RETN, and THBS1

SENSITIVITY (N = 9 ASSAYS)

| **Analyte** | **Minimum Detectable Concentration (pg/ml)** | **Minimum Detectable Concentration + 2 S.D.** |
| --- | --- | --- |
| ELA2 | 3 | 6.2 |
| LTF | 195 | 255 |
| LCN2 | 15 | 23 |
| RETN | 4.3 | 8.5 |
| THBS1 | 69.4 | 133.4 |

INTRA- AND INTER-ASSY % COEFFICIENTS OF VARIATION (%CV)

| **Analyte** | **Intra-assay %CV** | **Inter-assay %CV** |
| --- | --- | --- |
| ELA2 | 6 | 7 |
| LTF | 6 | 8 |
| LCN2 | 6 | 4 |
| RETN | 6 | 9 |
| THBS1 | 6 | 9 |

SPIKE RECOVERY IN SERUM MATRIX SOLUTION

| **Analyte** | **% Recovery in Serum Matrix** |
| --- | --- |
| ELA2 | 76 |
| LTF | 99 |
| LCN2 | 96 |
| RETN | 71 |
| THBS1 | 70 |
