# Supplementary material for: The pediatric sepsis biomarker risk model
Source: Crit Care. 2012 Oct 1;16(5):R174. doi: 10.1186/cc11652 (PMC3682273; doi:10.1186/cc11652)
Supplement: Additional File 2 — Derivation of the classification tree using Salford Predictive Modeler v6.6. This file provides the model parameters, pruning criteria, and the command file for generating the decision tree. [file cc11652-S2.DOC]

**Additional File 2**

**Derivation of the classification tree using Salford Predictive Modeler v6.6**

Testing Method: V-fold cross-validation (V = 10, default).

Priors: LEARN; Probabilities match learn sample frequencies.

Costs: 1.7; Dead misclassified as Survived.

Terminal node minimum cases: 5

Constraints: Disallowed a CCL3- or CCL4-dependent split beyond a depth of 2; disallowed an IL-8- or a LTF-dependent split beyond a depth of 4.

All other parameters remained as default settings.

Pruning rules:

1. At least one of two paired terminal daughter nodes contains ≥5% of the subjects in the root node.
2. No predictor variable could be repeated within one of the two main tree branches.

**Command file details for derivation of the classification tree using Salford Predictive Modeler v6.6**

REM***Resetting Preferences

REM***Setting General default options

LOPTIONS MEANS = NO, PREDICTIONS = NO, TIMING = NO, GAINS = NO, ROC = NO, PLOTS = NO

FORMAT = 5

REM***Setting CART default options

LOPTIONS, NOPRINT = NO, PS = NO

BOPTIONS SURROGATES = 5 PRINT = 5, COMPETITORS = 5 CPRINT = 5, TREELIST = 10,

BRIEF

SEED 13579, 12345, 131, NORETAIN

USE "C:\Users\WONO4W\Documents\H drive contents\PERSEVERE\Derivation cohort data\TEST of CART MODEL for COMPLICATED RECOVERY\Working files for metadata table\Biomarker Data for Salford.xls" ENCODING=SHIFTJIS

REM ***Setting General options

LOPTIONS MEANS = NO, PREDICTIONS = NO, TIMING = NO, GAINS = NO, ROC = NO, PLOTS = NO

FORMAT = 5

REM***Setting CART options

LOPTIONS, NOPRINT = NO, PS = NO

BOPTIONS SURROGATES = 5 PRINT = 5, COMPETITORS = 5 CPRINT = 5, TREELIST = 10,

BRIEF

LIMIT MINCHILD = 5

BOPTIONS MISSING = NO

DISCRETE MISSING = MISSING

CATEGORY

AUXILIARY

MODEL PATIENT_SURVIVE$

KEEP

KEEP GRANZYME_B, HSP70, IL_1A, IL_8, MIP_1A, MIP_1B, MMP8, ELASTASE, LACTOFERRIN,

NGAL, RESISTIN, THROMBOSPONDIN_1, GENDER$, AGE__YEARS_

LOPTIONS UNS = NO

CATEGORY PATIENT_SURVIVE$

AUXILIARY PRISM_SCORE

PRIORS LEARN

Misclassify Cost = 1.7 Classify "Dead" as "Survived"

DISALLOW

DISALLOW IL_8 / BELOW = 4 SPLIT

DISALLOW LACTOFERRIN / BELOW = 4 SPLIT

DISALLOW MIP_1A / BELOW = 2 SPLIT

DISALLOW MIP_1B / BELOW = 2 SPLIT

METHOD GINI POWER = 0.0000

BUILD

CATEGORY

AUXILIARY

MODEL PATIENT_SURVIVE$

KEEP

KEEP GRANZYME_B, HSP70, IL_1A, IL_8, MIP_1A, MIP_1B, MMP8, ELASTASE, LACTOFERRIN,

NGAL, RESISTIN, THROMBOSPONDIN_1, GENDER$, AGE__YEARS_

LOPTIONS UNS = NO

CATEGORY PATIENT_SURVIVE$

AUXILIARY PRISM_SCORE

DISALLOW

DISALLOW IL_8 / BELOW = 4 SPLIT

DISALLOW LACTOFERRIN / BELOW = 4 SPLIT

DISALLOW MIP_1B / BELOW = 2 SPLIT

METHOD GINI POWER = 0.0000

BUILD

CATEGORY

AUXILIARY

MODEL PATIENT_SURVIVE$

KEEP

KEEP GRANZYME_B, HSP70, IL_1A, IL_8, MIP_1A, MIP_1B, MMP8, ELASTASE, LACTOFERRIN,

NGAL, RESISTIN, THROMBOSPONDIN_1, GENDER$, AGE__YEARS_

LOPTIONS UNS = NO

CATEGORY PATIENT_SURVIVE$

AUXILIARY PRISM_SCORE

DISALLOW

DISALLOW IL_8 / BELOW = 4 SPLIT

DISALLOW LACTOFERRIN / BELOW = 4 SPLIT

METHOD GINI POWER = 0.0000

BUILD

CATEGORY

AUXILIARY

MODEL PATIENT_SURVIVE$

KEEP

KEEP GRANZYME_B, HSP70, IL_1A, IL_8, MIP_1A, MIP_1B, MMP8, ELASTASE, LACTOFERRIN,

NGAL, RESISTIN, THROMBOSPONDIN_1, GENDER$, AGE__YEARS_

LOPTIONS UNS = NO

CATEGORY PATIENT_SURVIVE$

AUXILIARY PRISM_SCORE

DISALLOW

DISALLOW IL_8 / BELOW = 4 SPLIT

DISALLOW LACTOFERRIN / BELOW = 4 SPLIT

DISALLOW MIP_1A / BELOW = 2 SPLIT

DISALLOW MIP_1B / BELOW = 2 SPLIT

METHOD GINI POWER = 0.0000

BUILD

CATEGORY

AUXILIARY

MODEL PATIENT_SURVIVE$

KEEP

KEEP GRANZYME_B, HSP70, IL_1A, IL_8, MIP_1A, MIP_1B, MMP8, ELASTASE, LACTOFERRIN,

NGAL, RESISTIN, THROMBOSPONDIN_1, GENDER$, AGE__YEARS_

LOPTIONS UNS = NO

CATEGORY PATIENT_SURVIVE$

AUXILIARY PRISM_SCORE

DISALLOW

DISALLOW IL_8 / BELOW = 4 SPLIT

DISALLOW MIP_1A / BELOW = 2 SPLIT

DISALLOW MIP_1B / BELOW = 2 SPLIT

METHOD GINI POWER = 0.0000

BUILD

CATEGORY

AUXILIARY

MODEL PATIENT_SURVIVE$

KEEP

KEEP GRANZYME_B, HSP70, IL_1A, IL_8, MIP_1A, MIP_1B, MMP8, ELASTASE, LACTOFERRIN,

NGAL, RESISTIN, THROMBOSPONDIN_1, GENDER$, AGE__YEARS_

LOPTIONS UNS = NO

CATEGORY PATIENT_SURVIVE$

AUXILIARY PRISM_SCORE

DISALLOW

DISALLOW LACTOFERRIN / BELOW = 4 SPLIT

DISALLOW MIP_1A / BELOW = 2 SPLIT

DISALLOW MIP_1B / BELOW = 2 SPLIT

METHOD GINI POWER = 0.0000

BUILD

CATEGORY

AUXILIARY

MODEL PATIENT_SURVIVE$

KEEP

KEEP GRANZYME_B, HSP70, IL_1A, IL_8, MIP_1A, MIP_1B, MMP8, ELASTASE, LACTOFERRIN,

NGAL, RESISTIN, THROMBOSPONDIN_1, GENDER$, AGE__YEARS_

LOPTIONS UNS = NO

CATEGORY PATIENT_SURVIVE$

AUXILIARY PRISM_SCORE

DISALLOW

DISALLOW IL_8 / BELOW = 4 SPLIT

DISALLOW LACTOFERRIN / BELOW = 4 SPLIT

DISALLOW MIP_1A / BELOW = 2 SPLIT

DISALLOW MIP_1B / BELOW = 2 SPLIT

METHOD GINI POWER = 0.0000

BUILD
