# Supplementary material for: The pediatric sepsis biomarker risk model
Source: Crit Care. 2012 Oct 1;16(5):R174. doi: 10.1186/cc11652 (PMC3682273; doi:10.1186/cc11652)
Supplement: Additional File 3 — List of comorbidities in survivors for the derivation and test cohorts. This file contains Table S1, which provides a list of co-morbidities for patients (survivors) in the derivation and test cohorts. [file cc11652-S3.DOC]

**Additional File 3, Table S1:** List of comorbidities in survivors for the derivation and test cohorts.

| **Derivation Cohort (N)** | **Test Cohort (N)** |
| --- | --- |
| Developmental Delay (15) | Bone marrow transplantation (9) |
| Bone marrow transplantation (7) | Acute lymphocytic leukemia (6) |
| Unspecified congenital heart disease (6) | Developmental delay (4) |
| Acute lymphocytic leukemia (4) | Medulloblastoma (3) |
| Short gut syndrome (4) | Unspecified congenital heart disease (3) |
| Drowning (3) | Acute myeloid leukemia (2) |
| Liver transplant (3) | Down Syndrome (2) |
| Neuroblastoma (3) | Aplastic anemia (1) |
| Severe combined immune deficiency (3) | Chronic granulomatous disease (1) |
| Unspecified brain tumor (3) | Chronic lung disease (1) |
| Glycogen storage disease type 1 (2) | DiGeorge Syndrome (1) |
| Hemophagocytic lymphohistiocytosis (2) | End stage renal disease (1) |
| Down Syndrome (2) | Hepatoblastoma (1) |
| Mitochondrial disorder (2) | Hypoplastic left heart syndrome (1) |
| Subglottic stenosis (2) | IPEX Syndrome (1) |
| Aplastic anemia (1) | Liver and Bowel Transplant (1) |
| Atrial and ventricular septal defects (1) | Multi-visceral transplant (1) |
| Caustic ingestion (1) | Multiple congenital anomalies (1) |
| Chronic lymphopenia (1) | Nephrotic syndrome (1) |
| Cyclic Neutropenia (1) | Obstructed pulmonary veins (1) |
| Cri Du Chat syndrome (1) | Prader-Willi Syndrome (1) |
| End stage renal disease (1) | Sarcoma (1) |
| Heterotaxy (1) | Sickle cell disease (1) |
| Hydrocephalus (1) | Small bowel transplant (1) |
| Kidney transplant (1) | Trisomy 18 (1) |
| Langerhans cell histiocytosis (1) | Wilms tumor (1) |
| Liver failure (1) |  |
| Medulloblastoma (1) |  |
| Metaleukodystrophy (1) |  |
| Neuromuscular disorder (1) |  |
| Pallister Killian Syndrome (1) |  |
| Prader-Willi Syndrome (1) |  |
| Retinoblastoma (1) |  |
| Rhabdomyosarcoma (1) |  |
| Rhabdosarcoma (1) |  |
| Seizure disorder (1) |  |
| Sleep apnea (1) |  |
| Tracheal stenosis (1) |  |
| Traumatic brain injury (1) |  |
| Type 1 diabetes mellitus |  |
| Unspecified leukemia (1) |  |
| VATER Syndrome (1) |  |
