# Supplementary material for: The pediatric sepsis biomarker risk model
Source: Crit Care. 2012 Oct 1;16(5):R174. doi: 10.1186/cc11652 (PMC3682273; doi:10.1186/cc11652)
Supplement: Additional File 4 — Causative organisms for derivation and test cohorts. This file contains Table S2, which provides a list of causative organisms for patients in the derivation and test cohorts. [file cc11652-S4.DOC]

**Additional File 4, Table S2:** Causative organisms for derivation and test cohorts.

| **Organism** | **Derivation Cohort (#)** | **Test Cohort (#)** |
| --- | --- | --- |
| *Acinetobacter baumannii* | 1 | 0 |
| Adenovirus | 1 | 0 |
| *Bacteroides* species | 1 | 0 |
| *Candida* species | 4 | 0 |
| *Capnotyophaga jenuni* | 0 | 1 |
| Cytomegalovirus | 1 | 0 |
| *Enterobacter cloacae* | 3 | 4 |
| *Enterococcus faecalis* | 4 | 1 |
| *Escherichia coli* | 2 | 1 |
| Herpes simplex virus | 2 | 2 |
| Human metapneumovirus | 0 | 1 |
| Influenza A | 4 | 4 |
| *Klebsiella pneumoniae* | 9 | 2 |
| *Micrococcus* species | 0 | 1 |
| Mixed | 23 | 14 |
| *Moraxella catarrhalis* | 1 | 0 |
| *Neisseria meningitidis* | 9 | 4 |
| Parainfluenza | 1 | 0 |
| *Pseudomonas* species | 3 | 2 |
| *Serratia marcescens* | 0 | 1` |
| *Staphylococcus aureus* | 11 | 6 |
| *Streptococcus agalactiae* | 3 | 3 |
| *Streptococcus milleri* | 1 | 0 |
| *Streptococcus pneumoniae* | 7 | 5 |
| *Streptococcus pyogenes* | 17 | 2 |
| Unspecified gram negative rod | 5 | 5 |
| Unspecified gram positive cocci | 6 | 3 |
