# Supplementary material for: The pediatric sepsis biomarker risk model
Source: Crit Care. 2012 Oct 1;16(5):R174. doi: 10.1186/cc11652 (PMC3682273; doi:10.1186/cc11652)
Supplement: Additional File 6 — Updating the classification tree using Salford Predictive Modeler v6.6. This file provided the model parameters, pruning criteria, and the command file for generating the updated decision tree. [file cc11652-S6.DOC]

**Additional File 6**

**Updating the classification tree using Salford Predictive Modeler v6.6**

Testing Method: V-fold cross-validation (V = 10, default).

Priors: EQUAL; all categories have equal probability.

Method: Class probability

All other parameters remained as default settings.

Pruning rules:

1. At least one of two paired terminal daughter nodes contains ≥5% of the subjects in the root node.
2. No predictor variable could be repeated within one of the two main tree branches.

**Command file details for updating the classification tree using Salford Predictive Modeler v6.6**

REM***Resetting Preferences

REM***Setting General default options

LOPTIONS MEANS = NO, PREDICTIONS = NO, TIMING = NO, GAINS = NO, ROC = NO, PLOTS = NO

FORMAT = 5

REM***Setting CART default options

LOPTIONS, NOPRINT = NO, PS = NO

BOPTIONS SURROGATES = 5 PRINT = 5, COMPETITORS = 5 CPRINT = 5, TREELIST = 10,

BRIEF

SEED 13579, 12345, 131, NORETAIN

USE "C:\Users\WONO4W\Documents\H drive contents\PERSEVERE_CALIBRATION_March 2012\Data for Calibration_March 2012_355 patients.xlsx" ENCODING=SHIFTJIS

REM ***Setting General options

LOPTIONS MEANS = NO, PREDICTIONS = NO, TIMING = NO, GAINS = NO, ROC = NO, PLOTS = NO

FORMAT = 5

REM***Setting CART options

LOPTIONS, NOPRINT = NO, PS = NO

BOPTIONS SURROGATES = 5 PRINT = 5, COMPETITORS = 5 CPRINT = 5, TREELIST = 10,

BRIEF

CATEGORY

AUXILIARY

MODEL PATIENT_SURVIVE$

KEEP

KEEP AGE__YEARS_, CCL3, CCL4, ELA2, GENDER$, GRANZYME_B_, HSP70_, IL1A, IL8, LTN,

MMP8_, NGAL_, RESISTIN_, THBS1

LOPTIONS UNS = NO

CATEGORY PATIENT_SURVIVE$

AUXILIARY COMPLICATED_REC_1___1_ORGAN_FAILURE_AT_DAY_3_OR_DEATH_$,

COMPLICATED_REC_2___1_ORGAN_FAILURE_AT_DAY_7_OR_DEATH_,

COMPLICATED_REC_2___1_ORGAN_FAILURE_AT_DAY_7_OR_DEATH_$, DATE_OF_BIRTH$,

MEDICAL_ID$, PICU_ADMIT_DATE$, PRISM_SCORE$, RACE$, RESEARCH_ID$

FORCE

FORCE ROOT ON CCL3

FORCE LEFT ON HSP70_

METHOD PROB POWER = 0.0000

BUILD

CATEGORY

AUXILIARY

MODEL PATIENT_SURVIVE$

KEEP

KEEP AGE__YEARS_, CCL3, CCL4, ELA2, GENDER$, GRANZYME_B_, HSP70_, IL1A, IL8, LTN,

MMP8_, NGAL_, RESISTIN_

LOPTIONS UNS = NO

CATEGORY PATIENT_SURVIVE$

AUXILIARY COMPLICATED_REC_1___1_ORGAN_FAILURE_AT_DAY_3_OR_DEATH_$,

COMPLICATED_REC_2___1_ORGAN_FAILURE_AT_DAY_7_OR_DEATH_,

COMPLICATED_REC_2___1_ORGAN_FAILURE_AT_DAY_7_OR_DEATH_$, DATE_OF_BIRTH$,

MEDICAL_ID$, PICU_ADMIT_DATE$, PRISM_SCORE$, RACE$, RESEARCH_ID$, THBS1

METHOD PROB POWER = 0.0000

BUILD

CATEGORY

AUXILIARY

MODEL PATIENT_SURVIVE$

KEEP

KEEP AGE__YEARS_, CCL3, CCL4, ELA2, GENDER$, GRANZYME_B_, HSP70_, IL1A, IL8, LTN,

MMP8_, NGAL_, RESISTIN_

LOPTIONS UNS = NO

CATEGORY PATIENT_SURVIVE$

AUXILIARY COMPLICATED_REC_1___1_ORGAN_FAILURE_AT_DAY_3_OR_DEATH_$,

COMPLICATED_REC_2___1_ORGAN_FAILURE_AT_DAY_7_OR_DEATH_,

COMPLICATED_REC_2___1_ORGAN_FAILURE_AT_DAY_7_OR_DEATH_$, DATE_OF_BIRTH$,

MEDICAL_ID$, PICU_ADMIT_DATE$, PRISM_SCORE$, RACE$, RESEARCH_ID$, THBS1

FORCE

FORCE ROOT ON CCL3

FORCE LEFT ON HSP70_

METHOD PROB POWER = 0.0000

BUIL
