# Supplementary material for: The pediatric sepsis biomarker risk model
Source: Crit Care. 2012 Oct 1;16(5):R174. doi: 10.1186/cc11652 (PMC3682273; doi:10.1186/cc11652)
Supplement: Additional File 7 — Comparison of PERSEVERE and PRISM for predicting mortality in the combined derivation and test cohorts. This file contains Table S3, which compares the test characteristics of PERSEVERE and PRISM. [file cc11652-S7.DOC]

**Additional File 7, Table S3:** Comparison of PERSEVERE and PRISM for predicting mortality in the combined derivation and test cohorts.

|  | **Re-calibrated PERSEVERE** | **PRISM at Sensitivity = PERSEVERE** | **PRISM at Specificity = PERSEVERE** |
| --- | --- | --- | --- |
| **Number of Subjects** | 355 | 353^1^ | 353^1^ |
| **True Positives** | 38 | 37 | 29 |
| **True Negatives** | 233 | 120 | 234 |
| **False Positives** | 81 | 193 | 79 |
| **False Negatives** | 3 | 3 | 11 |
| **Sensitivity** | 93% (79 – 98) | 93% (79 – 98) | 73% (56 – 85) |
| **Specificity** | 74% (69 – 79) | 38% (33 – 44) | 75% (69 – 79) |
| **Positive Predictive Value** | 32% (24 – 41) | 16% (12 – 22) | 27% (19 – 36) |
| **Negative Predictive Value** | 99% (96 – 100) | 98% (92 – 99) | 96% (92 – 98) |
| **+Likelihood Ratio** | 3.6 (2.9 – 4.4) | 1.5 (1.3 – 1.7) | 2.3 (2.2 – 3.8) |
| **-Likelihood Ratio** | 0.1 (0.0 – 0.3) | 0.2 (0.1 – 0.6) | 0.4 (0.2 – 0.6) |
| **Area under the curve** | 0.883 | 0.798 | 0.798 |

^1^Two participants (1 survivor and 1 non-survivor) did not have PRISM scores recorded.
